# Restoration of biofuel production levels and increased tolerance under ionic liquid stress is enabled by a mutation in the essential *Escherichia coli* gene *cydC*

**DOI:** 10.1186/s12934-018-1006-8

**Published:** 2018-10-08

**Authors:** Thomas Eng, Philipp Demling, Robin A. Herbert, Yan Chen, Veronica Benites, Joel Martin, Anna Lipzen, Edward E. K. Baidoo, Lars M. Blank, Christopher J. Petzold, Aindrila Mukhopadhyay

**Affiliations:** 1 Biological Systems and Engineering Division, Lawrence Berkeley National Laboratory, Berkeley, CA 94720 USA; 2 Institute of Applied Microbiology - iAMB, Aachen Biology and Biotechnology - ABBt, RWTH Aachen University, 52074 Aachen, Germany; 3 Joint BioEnergy Institute, Lawrence Berkeley National Laboratory, Emeryville, CA 94608 USA; 4 Joint Genome Institute, Lawrence Berkeley National Laboratory, Walnut Creek, 94598 USA; 5 Environmental Genomics and Systems Biology Division, Lawrence Berkeley National Laboratory, Berkeley, CA 94720 USA

**Keywords:** Ionic liquids, [EMIM]OAc, [C_2_C_1_im]OAc, Biofuels, Adaptation, Laboratory evolution, Isopentenol, Limonene, Strain engineering, *cydC*, *Escherichia coli*

## Abstract

**Background:**

Microbial production of chemicals from renewable carbon sources enables a sustainable route to many bioproducts. Sugar streams, such as those derived from biomass pretreated with ionic liquids (IL), provide efficiently derived and cost-competitive starting materials. A limitation to this approach is that residual ILs in the pretreated sugar source can be inhibitory to microbial growth and impair expression of the desired biosynthetic pathway.

**Results:**

We utilized laboratory evolution to select *Escherichia coli* strains capable of robust growth in the presence of the IL, 1-ethyl-3-methyl-imidizolium acetate ([EMIM]OAc). Whole genome sequencing of the evolved strain identified a point mutation in an essential gene, *cydC*, which confers tolerance to two different classes of ILs at concentrations that are otherwise growth inhibitory. This mutation, *cydC*-*D86G*, fully restores the specific production of the bio-jet fuel candidate d-limonene, as well as the biogasoline and platform chemical isopentenol, in growth medium containing ILs. Similar amino acids at this position in *cydC*, such as *cydC*-*D86V*, also confer tolerance to [EMIM]OAc. We show that this [EMIM]OAc tolerance phenotype of *cydC*-*D86G* strains is independent of its wild-type function in activating the cytochrome bd-I respiratory complex. Using shotgun proteomics, we characterized the underlying differential cellular responses altered in this mutant. While wild-type *E. coli* cannot produce detectable amounts of either product in the presence of ILs at levels expected to be residual in sugars from pretreated biomass, the engineered *cydC*-*D86G* strains produce over 200 mg/L d-limonene and 350 mg/L isopentenol, which are among the highest reported titers in the presence of [EMIM]OAc.

**Conclusions:**

The optimized strains in this study produce high titers of two candidate biofuels and bioproducts under IL stress. Both sets of production strains surpass production titers from other IL tolerant mutants in the literature. Our application of laboratory evolution identified a gain of function mutation in an essential gene, which is unusual in comparison to other published IL tolerant mutants.
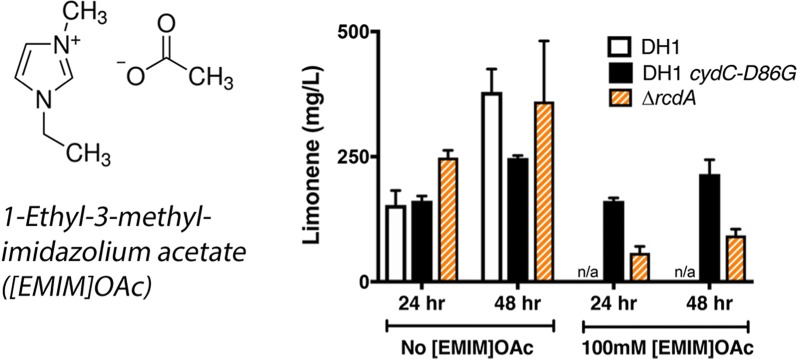

**Electronic supplementary material:**

The online version of this article (10.1186/s12934-018-1006-8) contains supplementary material, which is available to authorized users.

## Background

To enable the widespread adoption of next-generation biofuels, microbial hosts must be evaluated for productivity under cost-effective production regimes that can compete in the commodity market. Lignocellulosic plant biomass is a promising renewable carbon source, but the solubilization of sugars from this recalcitrant biomass is a technical challenge [1, 2]. One promising solution to this problem involves pretreating the plant biomass with an ionic liquid (IL), which allows the rapid extraction of sugars with greater ease when compared with conventional methods [3]. The resulting sugars from this pretreatment are then used in a microbial conversion process to produce the desired target compound. However, trace concentrations of many commonly used ILs from the lignocellulosic-derived carbon are inhibitory to cell growth [4–6], thus necessitating additional costly wash steps to fully purify this sugar source [7]. Moreover, unlike ethanol, the expression of next-generation biofuels from synthetic gene pathways requires additional optimization for function in the presence of ILs [8]. Optimization of both tolerance to ILs, as well as productivity under these growth constraints is required to fully exploit the value of biomass pretreated with ILs. One current approach is to develop ILs with low downstream impacts on the microbial conversion process [6, 9–11]. However, another useful strategy has also been to generate microbial hosts tolerant to a broad range of ILs, simplifying downstream microbial conversion of carbon sources to the target compounds.

The development of efficient microbial conversion in the presence of residual ILs remains a challenge in the field. Previous studies in this area have seen limited success in optimizing components of the process in a piecemeal fashion, as they achieve robust stress tolerance or high production, but not both. *Saccharomyces cerevisiae* has been used with IL pretreated biomass, but for the production of fermentation products such as ethanol [12]. With regards to advanced biofuels such as d-limonene and bisabolene [13], reports from the bacterial host, *E. coli,* have described more relevant advances. For example, Ruegg et al. used a gene cassette from an IL tolerant bacterium *Enterobacter lignolyticus* to auto-regulate expression of an imidazolium responsive efflux pump, which when expressed in *Escherichia coli*, improved host strain viability and allowed production of the biodiesel precursor bisabolene in the presence of the ionic liquid [14]. An adaptive laboratory evolution study to generate IL tolerant strains of *E. coli* has resulted in the discovery of several cellular transporters [15], which may prove to be useful in engineering production strains in future studies. Frederix et al. expressed IL tolerant cellulases in yet another IL tolerant *E. coli* background to use unsaccharified cellulose as a carbon source. The same strain, in turn, produced d-limonene (hereafter referred to as limonene), a bio-jet fuel, in the presence of ILs [16]. While successful relative to that in wild-type microbial strain backgrounds, the production of either bisabolene or limonene from Ruegg et al. or Frederix et al. was less than the maximum level produced by the same strain under optimal conditions. These results suggest that the IL inhibition of the biosynthetic pathways had not been fully addressed. Thus, it remains a challenge to develop a microbial strain tolerant to residual ILs used to pretreat biomass and still maintain robust bioconversion pathway productivity.

In this study, we used laboratory evolution to identify mutants that confer tolerance to the most commonly used model IL, 1-ethyl-3-methyl-imidizolium acetate (hereafter referred to as [EMIM]OAc). [EMIM]OAc has many favorable characteristics that facilitate the treatment and processing of biomass for sugar extraction by a variety of methods [17]. Whole genome re-sequencing implicated *cydC*, a cytochrome assembly factor and component of an ABC transporter complex [18] as the candidate gene for the observed tolerance. We successfully produced two candidate biofuels (limonene and isopentenol) in the presence of [EMIM]OAc and characterized global proteomic changes that occur under [EMIM]OAc stress.

## Results

### Laboratory evolution identifies *cydC*-*D86G*, a mutant that confers tolerance to [EMIM]OAc

**Fig. 1:**
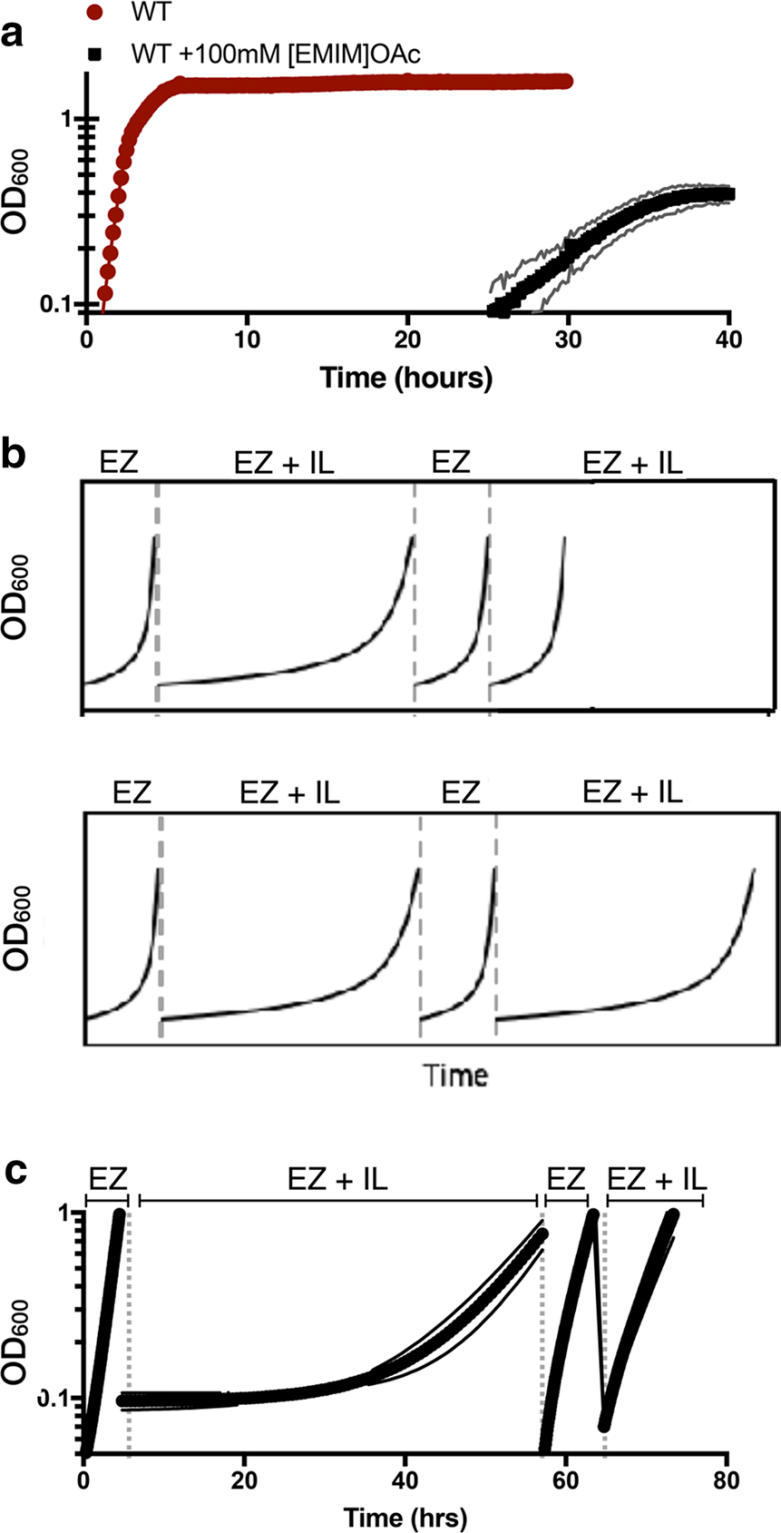
Adaptation and Laboratory Evolution of *E. coli DH1* in presence of [EMIM]OAc. **a** Exponential phase *E. coli* cells were grown in the presence of 100 mM [EMIM]OAc at 30 °C in a microtiter dish until a change in optical density was detected (“Methods”). *Red circles*, growth of wild-type cells in the absence of [EMIM]OAc. *Black squares*, growth of wild-type cells in the presence of [EMIM]OAc. Standard deviation from three biological replicates is indicated as the shaded region surrounding the red circles or black squares. Optical density is plotted on a log_10_ scale. **b** Schematic, behavior of cells after gaining a heritable mutation (top panel) compared to adaptation (bottom panel). Cells initially grown in the absence of the ionic liquid stress (“EZ”) are back-diluted into medium containing the ionic liquid (“EZ + IL”). After a long lag phase, an increase in optical density is detected. Cells are subsequently back diluted into fresh media without the ionic liquid (“EZ”) and then retested for growth in the presence of the ionic liquid (“EZ + IL”). A heritable mutation would prevent the appearance of an extended lag phase after the first exposure to an ionic liquid. If *E. coli* adapts to the IL, cells would show a similarly long lag phase as with the first treatment with ionic liquid. **c** Cells exhibit a heritable response to [EMIM]OAc. All three independent isolates show similar tolerance to [EMIM]OAc. Error bars indicate standard deviation from three biological replicates

We identified an improved host chassis for tolerance to [EMIM]OAc using laboratory evolution while maintaining production conditions. Mutant strains in a wild-type *E. coli* background that were capable of growing in 100 mM [EMIM]OAc were recovered after only 48 h of selection with exogenous [EMIM]OAc (Fig. 1a). This concentration of [EMIM]OAc was selected because it represents a typical concentration of trace IL after three washes with water, as opposed to the full eight water/ethanol wash regimen required to reduce residual IL levels below detection levels [19]. In order to validate that these candidate mutants were genetic mutations and not the result of adaptation, we used a dynamic cultivation regime [20] to alternate rounds of growth in the presence or absence of [EMIM]OAc (Fig. 1b, c). Five different isolates from two different rounds were recovered from this screen, and strikingly, three of them had similar robust growth profiles when compared side by side (Fig. 1c).

To identify potential putative mutations, cells from the liquid cultures were streaked to single colonies on solid agar plates, and a single colony from each plate was selected at random to confirm tolerance to the IL. We generated genomic libraries of all five mutants recovered from this study. Libraries were subjected to MiSeq Illumina deep sequencing. Basepairs that differed from the parent strain were called with CNVnator [21] (“Methods”). As these spontaneous mutants were derived from independent liquid cultures started from independent single colonies, we expected to find different mutants in different chromosomal loci that led to similar phenotypes. Remarkably, a single hitherto unknown mutation was identified in all five libraries. The mutation identified across all five of these isolates, but absent in the parental strain, was an aspartic acid to glycine mutation at residue 86 of *cydC*, a membrane protein thought to be involved in cytochrome bd-I assembly or amino acid efflux into the periplasm [22–26]. There were conflicting reports regarding *cydC* being an essential or dispensable gene. Without this knowledge, it could not be deduced directly whether *cydC*-*D86G* was a loss-of-function or gain-of-function mutation [27, 28]. In the two IL-tolerant, but slower growing mutant strains, we also detected unique premature stop codons (amber mutations) in the open reading frame of *rcdA.* This is consistent with a *rcdA* mutation identified in a previous study, which could ameliorate IL toxicity but did not fully restore the limonene production titer [16]. The amino acid change recovered in *cydC* was a strong candidate for the increased tolerance to the model IL, [EMIM]OAc, and belongs to a different functional category from IL tolerance genes discovered thus far. To assess the benefits of both improved tolerance and production, we focused our study on understanding this mutant.

**Fig. 2:**
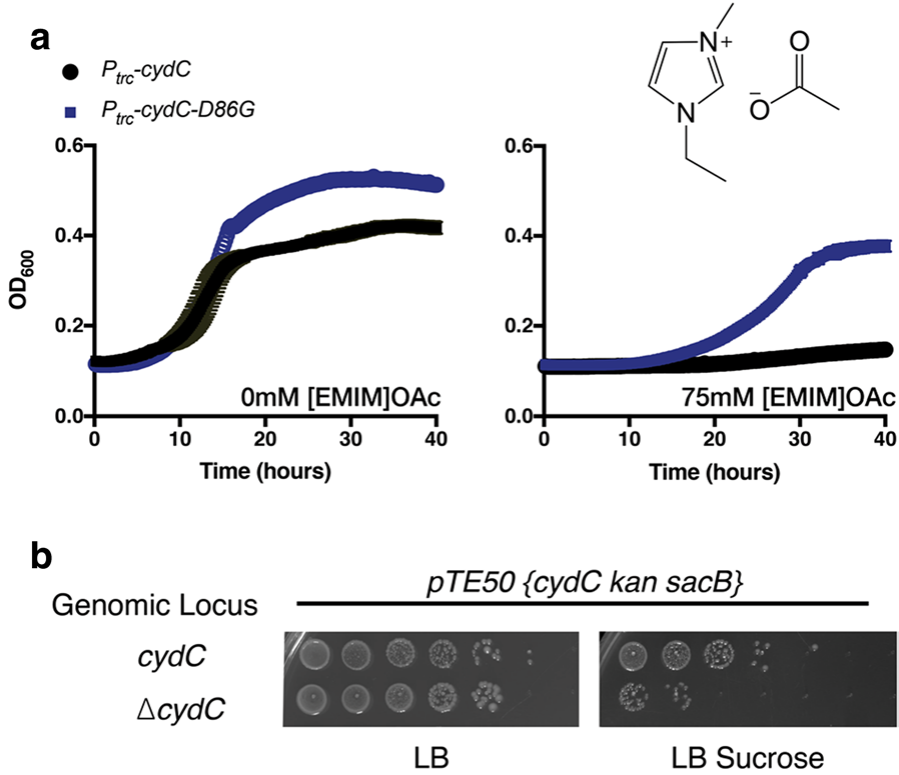
*cydC* Is An Essential Gene, and Expression of *cydC*-*D86G* is sufficient to confer tolerance to [EMIM]OAc. **a** *E. coli* DH1 cells harboring a plasmid-borne copy of either wild type or mutant *cydC*-*D86G* [pTE42*/P*_*trc*_-*cydD*-*cydC* or pTE43/*P*_*trc*_-*cydD*-*cydC*-*D86G*] were untreated or exposed to 75 mM [EMIM]OAc (“Methods”). The change in OD_600_ was measured every fifteen minutes for 40 h. **b** Evidence that *cydC* is essential by plasmid shuffle assay. After generating a strain with both plasmid pTE50 and ∆*cydC*, cells are grown without selection to allow for spontaneous plasmid loss. The rate of plasmid loss in both wild type and ∆*cydC* strains is compared by selecting for cells without the plasmid on sucrose plates. To allow for spontaneous plasmid loss, cells were grown under non-selective conditions for 24 h at 30 °C. Cells were serially diluted after growth in nonselective media, and plated onto solid LB agar media with or without 10% sucrose. Colonies that are viable in the presence of sucrose have lost the plasmid

We first examined the effect of this mutation on microbial growth. To confirm that it is the mutant *cydC*-*D86G* that conferred tolerance to [EMIM]OAc, we constructed plasmids encoding wild-type *cydC* or the mutant, *cydC*-*D86G,* under the control of the inducible *trc* promoter and transformed these plasmids into the parental strain. Since concentrations higher than 4 µM IPTG resulted in growth inhibition when overexpressing wild-type *P*_*TRC*_-*cydC* (Additional file 1: Figure S1), subsequent experiments relied on leaky gene expression without addition of the inducer. Upon treatment with [EMIM]OAc, the strain carrying an additional wild-type copy of *P*_*TRC*_-*cydC* failed to grow when treated with 75 mM [EMIM]OAc (Fig. 2a). In contrast, the strain carrying an additional copy of *P*_*TRC*_-*cydC*-*D86G* was viable in the presence of 75 mM [EMIM]OAc (Fig. 2a). In addition to conferring tolerance to [EMIM]OAc, we also observed that the original DH1 *cydC*-*D86G* strains were also somewhat tolerant to a structurally different IL, ethanolamine acetate ([EOA]OAc Additional file 1: Figure S2).

To maximize the ease of deploying this IL-tolerant gene to other strain backgrounds, we mutagenized the *cydC*-*D86* codon and screened for additional point mutants which were also tolerant to [EMIM]OAc. This time, however, we used the native *cydC* promoter. Increased expression of *cydC* under its native promoter from a multi-copy plasmid had no impact on cell growth, corroborating reports from the literature [24]. From this screen we identified that *cydC*-*D86V* was also tolerant to [EMIM]OAc. As we did not modify the background genomic copy of *cydC* in these cells, this implied that the plasmid-borne *cydC*-*D86V* still conferred tolerance to [EMIM]OAc, even when wild-type *cydC* was potentially expressed in the cell. These results indicate that the *cydC*-*D86G* mutant by itself is sufficient to confer tolerance to [EMIM]OAc as well as to a lesser extent [EOA]OAc, and that this activity is distinct from an increased expression of the wild-type *cydC* gene. Furthermore, we identified that similar point mutants such as *cydC*-*D86V* can also confer tolerance to [EMIM]OAc. This allows for rapid deployment of the *cydC* mutant versions with a simple plasmid transformation, without requiring laborious genomic allele replacement strategies.

We also confirmed that the single point mutation (D86G) in *cydC* specifically provides the IL tolerance. One mechanism for the new function is that *cydC*-*D86G* encodes a loss-of-function mutation and thus inactivated *cydC* is responsible for the observed phenotype. A loss-of-function mutation can only be expected in genes that are not essential, since essential genes cannot be deleted. However, reports in the literature do not allow a clear assignment of this aspect for *cydC*. Gene deletions of *cydC* are absent from *E. coli* deletion collections [27, 29] suggesting that it is an essential gene required for *E. coli* viability. As such, a direct gene-deletion approach using a selectable drug cassette could increase the potential false positives (via spontaneous suppressor mutations) that would confound our analysis [30].

Thus, we used a plasmid shuffle strategy to determine whether *cydC*-*D86G* might represent a loss-of-function mutation. We generated strains that harbor the *cydCD* operon on a plasmid in a wild-type background, and separately, in a background with a precise deletion of the *cydC* locus (“Methods”). We then screened for spontaneous plasmid loss using the plasmid-borne counterselection gene *sacB* [31] (Fig. 2b). Wild-type cells lost a plasmid-borne copy of *cydC* at a rate five orders of magnitude higher than the ∆*cydC* strains, indicating that strains without functional *cydC* are not favored (Fig. 2b). As CydC is functional only when in a heterodimeric complex with its partner protein, CydD [26], our result also suggests that previous studies examining ∆*cydC* or ∆*cydD* mutants need to be interpreted with caution, and that mutant alleles thought to be total loss-of-function alleles may be partially active [23, 32]. We conclude from this result that *cydC*-*D86G* could not be encoding a nonfunctional protein in the strain isolated in our study, and that *cydC*-*D86G* necessarily retains the activity required for cell viability equivalent to the wild-type gene. Thus, *cydC*-*D86G* must be a gain-of-function mutant. This conclusion is also consistent with our previous result, which indicated that overexpression of *cydC*-*D86G* conferred tolerance to [EMIM]OAc, but overexpression of the wild-type *cydC* alone did not confer tolerance to the IL in this background (refer to Fig. 2a).

### CydC does not improve IL tolerance via the cytochrome bd-I respiratory complex

**Fig. 3:**
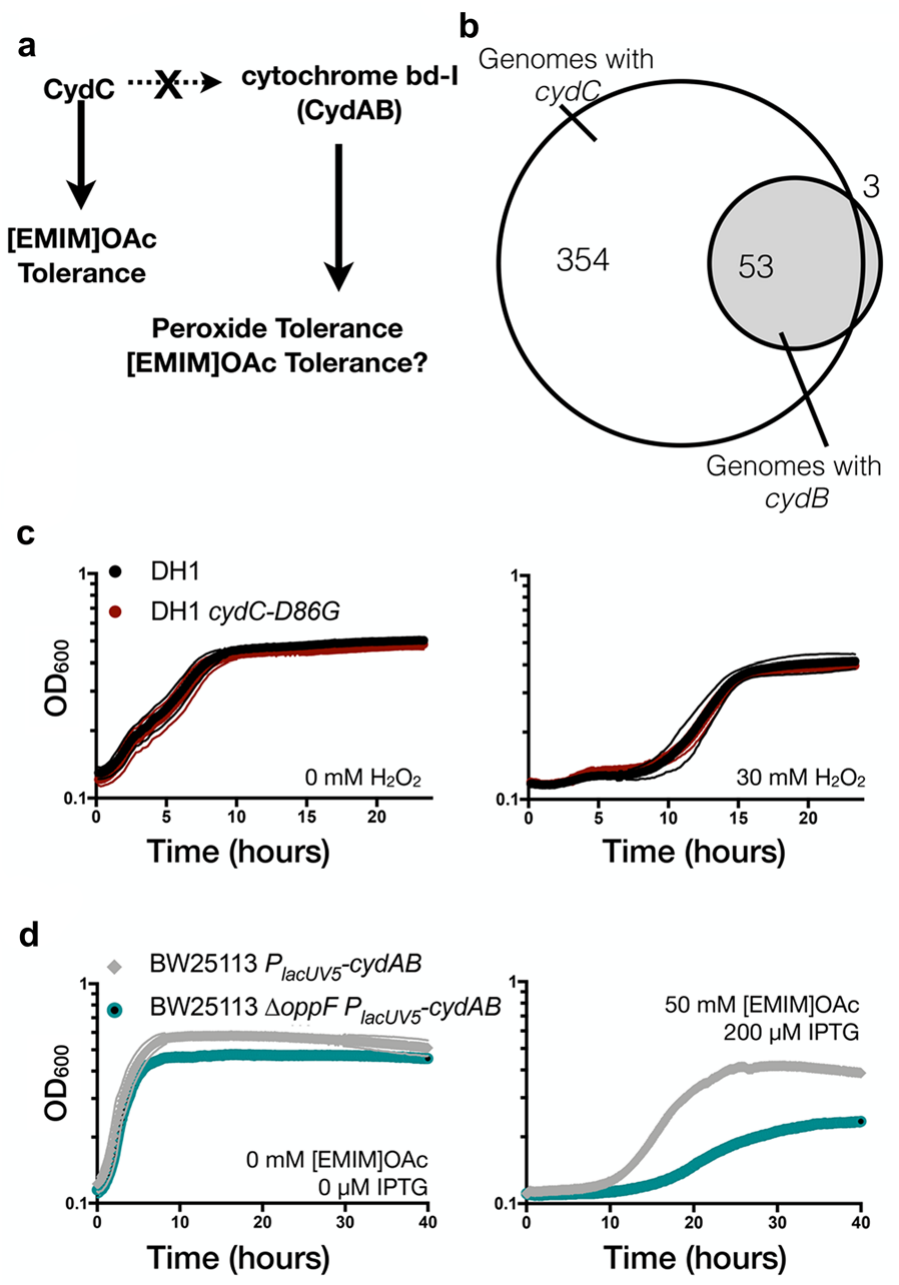
CydC Mediated [EMIM]OAc Tolerance Does Not Act Through cytochrome bd-I. **a** Schematic. Proposed relationship between CydC and cytochrome bd-I if [EMIM]OAc tolerance in CydC-D86G strains is due to directly activating cytochrome bd-I. **b** Conservation of *cydC* and cytochrome bd-I subunit *cydB* by strict (> 95% identity) homolog identification in bacteria. *cydB* is observed in a subset of species with detected *cydC* homologs. **c** Peroxide challenge assay. Wild-type DH1 cells or DH1 *cydC*-*D86G* cells grown in the presence of exogenous 30 mM hydrogen peroxide over a 20 h timeframe. No difference in hydrogen peroxide sensitivity was observed. Optical density (OD) is plotted on a log_10_ scale. **d** Tolerance to [EMIM]OAc in improved cytochrome bd-I cells. *E. coli* BW25113 ∆*oppF* [*P*_*trc*_-*cydAB*] or wild-type cells were grown in the presence of 50 mM [EMIM]OAc. Functional cytochrome bd-I activity was increased with 200 µM IPTG. Growth of cells was monitored over a 40 h timeframe. Optical density is plotted on a log_10_ scale

Next, we sought to understand the mechanistic basis of IL tolerance in the *E. coli cydC*-*D86G* strain. The lack of a crystal structure for *cydC* makes it challenging to assess the role of a point mutation, especially in a transmembrane domain. One possible mechanism by which *cydC* could confer tolerance to [EMIM]OAc as well as improve terpene production is through the activation of the cytochrome AB complex, as some studies have implicated the *cydCD* gene product in assembling functional cytochrome bd-I (CydAB) complexes [22]. Cytochrome bd-I is also known to be involved in limiting oxidative damage such as that caused by the detoxification of free oxygen radicals [33]. Consistent with this notion, studies in budding yeast and filamentous fungi have implicated [EMIM]OAc in respiratory impairment [34–36]. If cytochrome CD complexes play a role in activating cytochrome bd-I, this could result in an indirect mechanism for [EMIM]OAc tolerance. Logically, the hypothesis based on these assumptions is that increased cytochrome bd-I activity would have the same behavior as the *cydC*-*D86G* mutant (Fig. 3a).

To evaluate this hypothesis, we used available genomic data and a phylogenetic approach to survey whether *cydB* and *cydC* always co-occur in bacterial genomes [37]. If CydC activity is strictly required to activate cytochrome bd-I (CydAB*)*, it is likely that genomes with *cydB* would also encode *cydC*. This was not the case. Computational analysis identified highly conserved *cydC* homologs in 407 species, but only a subset of species (53 in total) contained a *cydB* homolog (Fig. 3b). Additionally, three isolates of *E. coli* encoded *cydB*, but did not contain a *cydC* homolog (Additional file 2: Table S1). These exceptions imply that *cydC* is not always required for *cydB* activity and furthermore, that *cydC* and *cydB* are not necessarily co-correlated in bacterial genomes.

The assumption that the function of *cydC*-*D86G* involves cytochrome bd-I activity also leads to two testable predictions. The first prediction is that *E. coli cydC*-*D86G* strains may have increased hydrogen peroxide resistance, the same phenotype as cells with increased cytochrome bd-I activity [38]. We subjected wild-type *E. coli* and *E. coli cydC*-*D86G* strains to increasing concentrations of exogenous hydrogen peroxide (“Methods”). We did not observe an increase in peroxide resistance in *E. coli cydC*-*D86G* cells, as both wild-type *E. coli* and *E. coli cydC*-*D86G* strains had the same sensitivity to exogenous hydrogen peroxide at concentrations up to 30 mM exogenous H_2_O_2_ (Fig. 3c). This observation is inconsistent with the hypothesis that *cydC*-*D86G* strains have more cytochrome bd-I complex activity. The second prediction is that strains with increased cytochrome bd-I activity may also have increased tolerance to the IL [EMIM]OAc. We tested this hypothesis using *E. coli* strains which we have previously shown to have increased cytochrome bd-I activity as measured by quantitatively improved hydrogen peroxide resistance [38]. This strain (*E. coli* ∆*oppF P*_*lacUV5*_-*cydAB*) was grown in the presence of [EMIM]OAc (Fig. 3d). We found that *cydAB* overexpression led to a longer doubling time and lower maximum growth (Fig. 3d). Induction of *cydAB* alone in the absence of the IL, or growth in the presence of the IL without induction of *cydAB* had no impact on growth (Additional file 1: Figure S3A, B). Additionally, we ruled out strain background effects by confirming that *cydC* and *cydC*-*D86G* confer IL tolerance in the related BW25113 strain background (Additional file 1: Figure S3C). The mutant protein CydC-D86G shows a function distinct from activating the cytochrome bd-I complex (Fig. 3a). Together these results indicate that strains with improved cytochrome bd-I activity are not tolerant to [EMIM]OAc, and do not recapitulate the IL tolerance that we observed in *cydC*-*D86G* strains.

### Identification of a cydC-mediated response network independent of cytochrome bd-I activation

Since *cydC*-*D86G* is a gain-of-function mutant and acts independently from cytochrome bd-I activation, we speculated that this new activity was related to its role in maintaining cytoplasmic homeostasis and alleviating nitrosative stress [23]. With FT-IR and HPLC-LCMS analysis, we ruled out the possibility that *cydC*-*D86G* strains were consuming or modifying the [EMIM]^+^ ion (Additional file 1: Figure S4). To examine if the mutant *cydC*-*D86G* strain is more effective than the wild-type strain at upregulating global stress regulators in response to [EMIM]OAc, we took a systems-level approach to ascertain the affected downstream proteins in *cydC*-*D86G* cells.

**Fig. 4:**
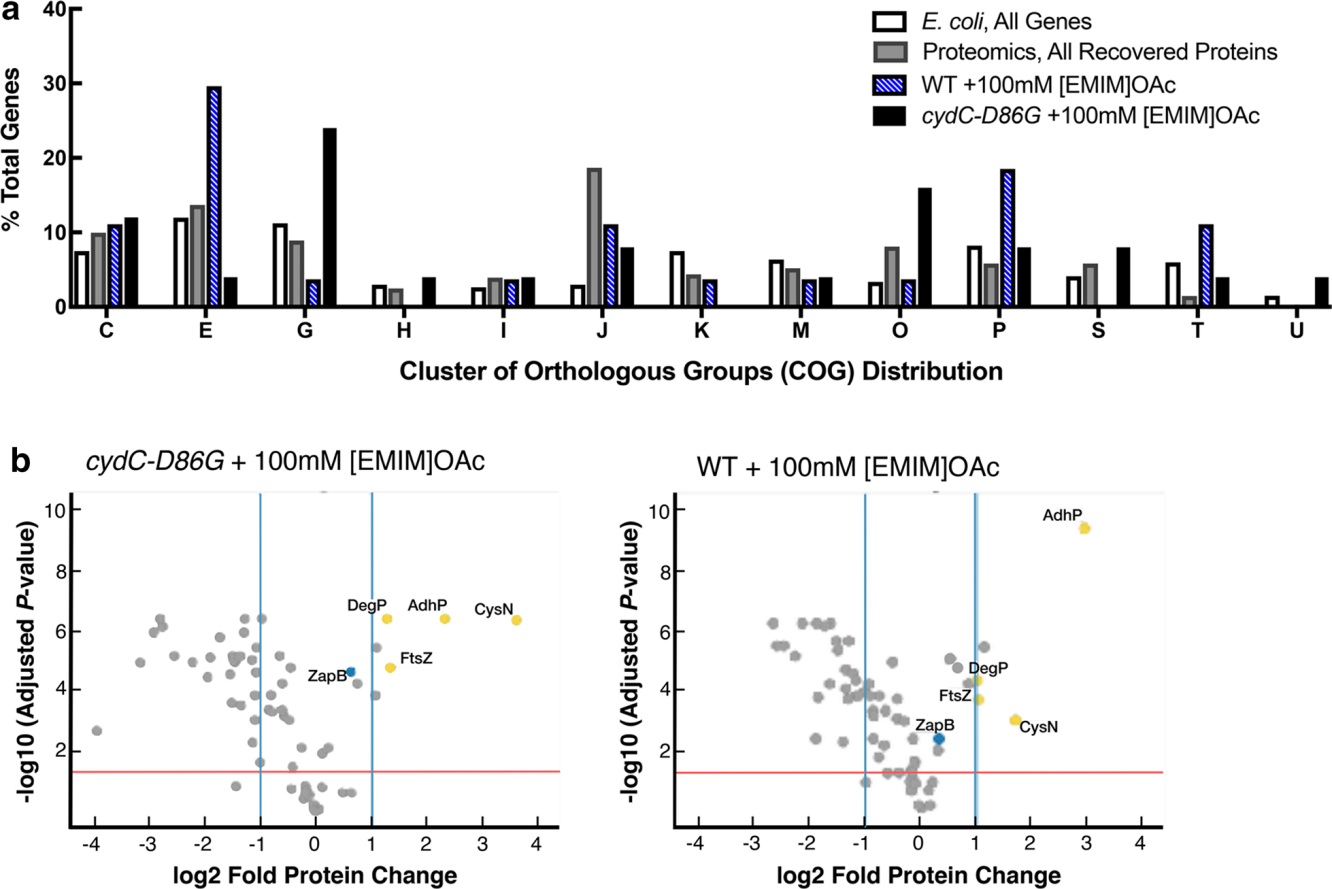
Shotgun Proteomics Identifies Enriched Proteins in *cydC*-*D86G* Cells Upon [EMIM]OAc Treatment. **a** Strains harboring the limonene production plasmid (JBEI-6409) and a plasmid-borne copy of *cydC*-*D86G* [pTE42/*P*_*trc*_-*cydD*-*cydC*-*D86G*] were prepared for whole genome proteomic analysis (“Methods”). Samples were harvested immediately before induction with 1 µM IPTG and 48 h after induction. Proteins at least 2 × enriched were categorized into functional categories (COG, Cluster of Orthologous Groups) (“Methods”). White columns; distribution of COG terms from the *E. coli* genome [29]. Gray columns; subset of recovered proteins from shotgun proteomics binned into COGs and plotted as the percent of all proteins recovered. Blue striped bars; COG distribution from DH1 treated with 100 mM [EMIM]OAc plotted as the percent of enriched proteins. Black bars; COG distribution from DH1 *cydC*-*D86G* treated with 100 mM [EMIM]OAc plotted as the percent of enriched proteins. **b** Candidate effector proteins enriched at least 2× were validated with quantitative peptide-area proteomics. The same experimental regimen and cells used in **a** were used to confirm protein abundance. The fold enrichment (log_2_) of unique peptides are plotted against their normalized significance (*P*) values. COG descriptions. [C], Energy production and conversion. [E], Amino Acid metabolism and transport. [G], Carbohydrate metabolism and transport. [H], Coenzyme metabolism. [I], Lipid metabolism. [J], Translation. [K], Transcription. [M], Cell wall/membrane/envelope biogenesis. [O], Post-translational modification, protein turnover, chaperone function. [P]. Inorganic ion transport and metabolism. [S], Function Unknown. [T], Signal Transduction. [U], Intracellular Trafficking and Secretion

Using shotgun proteomics, we compared samples with or without the *cydC*-*D86G* variant under typical production conditions (“Methods”) and measured the relative protein abundance levels for nearly 500 *E. coli* proteins. Samples from an empty vector control and the vector-borne *cydC*-*D86G* were harvested before treatment with [EMIM]OAc, 24 h after addition of the IL, and 48 h after addition of the IL. We identified proteins whose levels were increased or decreased at least twofold in the presence of [EMIM]OAc in *cydC*-*D86G* strains normalized to the protein levels before addition of [EMIM]OAc. The corresponding genes were binned into functional categories (COGs) and plotted against the overall gene abundances in the *E. coli* genome (Fig. 4a). Similar categorization was applied for all 486 proteins identified at high confidence to determine the selection bias from the analysis method compared to overall gene categories. We observed an enrichment of detected proteins related to “translation, ribosomal structure and biogenesis” (COG: J) when comparing total identified proteins to the *E. coli* genomic distribution. Due to their enrichment from the method alone, we considered these proteins to be false positives and did not pursue them further. However, we did detect enrichment of several gene categories that are specific to the increased IL tolerance in the *cydC*-*D86G* strains and which provide an improved understanding of IL tolerance in this mutant. Compared to wild-type cells and untreated *cydC*-*D86G* cells, genes in both “carbohydrate metabolism and transport (COG: G)” and “chaperone function, post translational modification, and protein turnover (COG: O)” categories were enriched when compared to the overall COG distribution relative to that in the wild type. The category “amino acid metabolism and transport (COG: E)” was also enriched in *cydC*-*D86G* strains treated with [EMIM]OAc, but in general the fold enrichment was higher in *cydC*-*D86G* strains then compared to the empty vector control. All identified genes and their enrichment values detected by this shotgun proteomics method are described in Additional file 3: Table S2.

To validate these increased or decreased protein candidates, we developed an MRM based targeted proteomic method (“Methods”), and relatively quantified them among treatment conditions [39]. Statistical significance was confirmed across these four biological replicates for each treatment condition (Fig. 4b). The degree of peptide enrichment for both wild type and *cydC*-*D86G* strains was similar but proteins such as AdhP and DegP showed differential levels of enrichment (Fig. 4b). All proteins detected by the MRM method and their calculated p-values are described in Additional file 4: Table S3. Together, these results indicate that *E. coli* cells respond to [EMIM]OAc stress by upregulating amino acid metabolism related genes, but *cydC*-*D86G* strains specifically upregulate two additional gene classes, namely chaperones and carbohydrate metabolism genes.

To understand the role of these candidates as downstream effectors of the *cydC*-*D86G* phenotype, we reasoned that single gene deletion mutants might show differences in their ability to handle [EMIM]OAc stress. If a deletion mutant was sensitive to [EMIM]OAc, it would imply that the gene was required for the altered *cydC*-*D86G* stress response. Alternatively, deletion strains that were more resistant to the IL could indicate that *cydC*-*D86G* dampened the gene activity. However, a negative result, i.e. deletion mutants which had no change in phenotype, are also likely and are expected to arise from redundant or overlapping effector pathways.

We selected 20 deletion strains among protein candidates potentially involved in *cydC*-*D86G* mediated resistance to this IL. Many of the strains tested had subtle colony phenotypes compared to wild-type *E. coli*, ∆*emrE*, or ∆*rcdA* cells, two published deletion strains with sensitivity or resistance to [EMIM]OAc, respectively [16, 40]. Of the targets tested, *E. coli* ∆*zapB* cells were sensitive to this IL (Table 1). ∆*adhP* cells were more resistant to this IL, consistent with its statistically significant decrease in abundance in *cydC*-*D86G* strains (Table 1). ZapB is a non-essential protein which associates with FtsZ and is important for efficient chromosomal segregation [41]. The connection between increased ZapB activity and the role of *cydCD* complexes in this phenotype is unclear, but data for this candidate suggest that it may be a key response factor influenced by *cydC* activity in mediating tolerance to this IL. Essential genes such as *plsB* could not be examined. Overall, these results imply that while no single downstream gene is fully attributable for the *cydC*-*D86G* phenotype, specific categories of functions, chaperones, and carbohydrate metabolism can be implicated in the corresponding IL tolerance.

**Table 1:** Evaluation of [EMIM]OAc sensitivity in *E. coli* single gene deletion strains

Mutant	JW accession number	Viability, LB 100 mM [EMIM]OAc
BW25113		+
∆*acnA*	JW1268	+
∆*adhP*	JW1474	+ +
∆*cysH*	JW2732	+
∆*cysI*	JW2733	+
∆*cysN*	JW2721	+
∆*emrE*	JW0531	+/−
∆*fbaB*	JW5344	+/−
∆*grxB*	JW1051	+
∆*katE*	JW1721	+
∆*manX*	JW1806	+
∆*nuoC*	JW5375	+
∆*osmC*	JW1477	+
∆*pspA*	JW1297	+
∆*pykA*	JW1843	+
∆*rcdA*	JW5114	+ + +
∆*slp*	JW3474	+/−
∆*ugpB*	JW3418	+
∆*uspG*	JW0600	+
∆*ybgL*	JW0703	+
∆*ydiH*	JW1675	+
∆*yihL*	JW3837	+
∆*zapB*	JW3899	−

Deletion mutants corresponding to candidate upregulated genes identified from shotgun proteomics were obtained from the Keio collection. Precultures of the indicated genotype were serially diluted into wells of a microtiter plate and spotted onto solid LB agar media with or without 100 mM [EMIM]OAc. Growth of each *E. coli* deletion strain was compared to growth of a wild-type *E. coli* of the same strain background (BW25113). No pre-existing growth defects were observed on LB agar plates without supplemental [EMIM]OAc

### *cydC*-*D86G* strains produce high titers of limonene or isopentenol under [EMIM]OAc stress

**Fig. 5:**
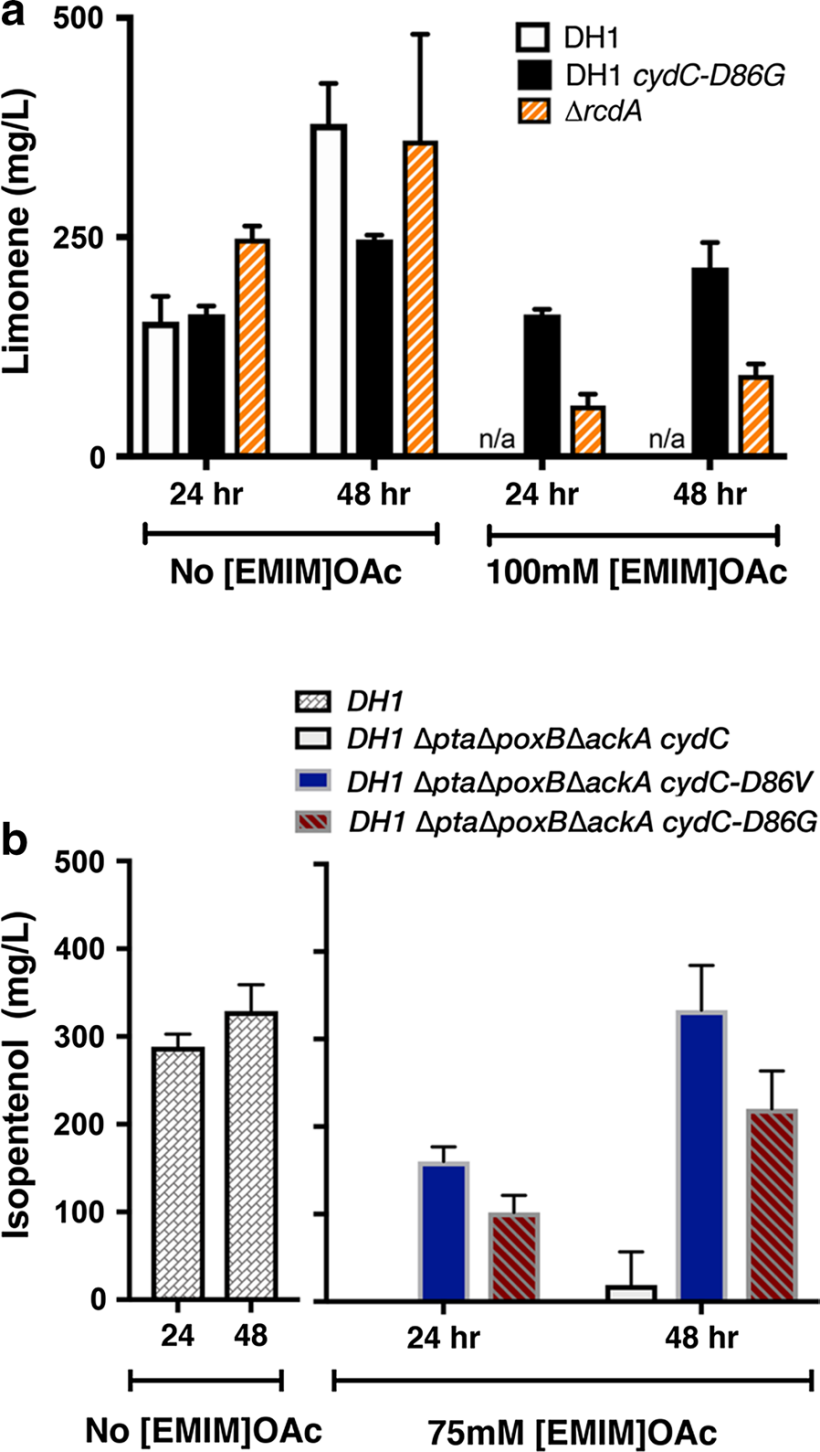
Production of the Biofuel Precursor Limonene and Isopentenol Under Exogenous [EMIM]OAc Growth Conditions. **a** *E. coli* DH1 cells with or without the *cydC*-*D86G* mutation at the genomic locus and a limonene production plasmid (JBEI-6409) were tested for limonene production in the presence or absence of 100 mM [EMIM]OAc (“Methods”). Time points were sampled 24 h or 48 h after pathway induction with 15 µM IPTG. Black bars; WT strains. Grey bars; *cydC*-*D86G* strains. Orange bars; ∆*rcdA* strains. **b** *E. coli* DH1 ∆*pta*∆*poxB*∆*ackA* (JBEI-3606) harboring [pTE50/*P*_*cydD*_-*cydD*-*cydC*] (white bars), [pTE88*/P*_*cydD*_-*cydD*-*cydC*-*D86G*] (red striped bars), or [pTE100 *P*_*cydD*_-*cydD*-*cydC*-*D86V*] (blue bars) along with an isopentenol production plasmid (JBEI-9321) were prepared for biofuel production in the presence of 75 mM [EMIM]OAc. Sampling was conducted at 24 h or 48 h after pathway induction with 500 µM IPTG. Production of isopentenol was compared to *E. coli* DH1 strains harboring an empty plasmid (*P*_*cydD*_ only) and the isopentenol production plasmid (JBEI-9321) in the absence of [EMIM]OAc. Only trace isopentenol production (with high acetate formation) was observed in the presence of [EMIM]OAc using either *E. coli* DH1 or *E. coli* DH1 *cydC*-*D86G* strains (data not shown)

As a cytochrome assembly factor or an amino acid transporter, *cydC* represents a different functional group from most tolerance mechanisms discovered to date. To test how well *cydC*-*D86G* strains produced limonene under [EMIM]OAc stress conditions, we cultivated *cydC*-*D86G* strains harboring the limonene production plasmid (JBEI-6409) [42] in the presence of 100 mM [EMIM]OAc. Strains harboring either wild-type *cydC* or *cydC*-*D86G* at the *cydC* genomic locus were examined for limonene production. Samples were collected 24 and 48 h after induction of the limonene production pathway with 15 µM IPTG. We observed that, unlike previously discovered IL tolerant strains, the *cydC*-*D86G* strain was an excellent limonene producer in the presence of 100 mM [EMIM]OAc. The *cydC*-*D86G* strain reached titers of above 200 mg/L limonene in 48 h, and a specific production of 80 mg/L/OD (Fig. 5a, Additional file 1: Figure S5), equivalent to the limonene production in the absence of IL in any of the strains tested. In contrast, the ∆*rcdA* strain only partially restored production under these cultivation parameters (Fig. 5a). Wild-type *E. coli* cells carrying only the limonene production plasmid, or an additional copy of wild-type *cydC* failed to grow in the presence of [EMIM]OAc and could not be tested for limonene production (Fig. 5a). We conclude from these results that the *cydC*-*D86G* strain has two advantages: it increases tolerance to exogenous [EMIM]OAc and restores high titers of limonene in the presence of this IL.

To examine if the improvement in production was specific to limonene as a final product, we tested the production of another biofuel candidate and platform chemical derived from the mevalonate pathway, 3-methyl-3-buten-1-*ol*, (hereafter referred to as isopentenol) [43, 44]. When we assayed the initial isopentenol production strain which carried both the isopentenol production pathway as well as *cydC*-*D86G* on a second plasmid, only trace amounts of isopentenol were detected by GC–FID when cultured in the presence of exogenous [EMIM]OAc. Instead, the majority of the product was acetate. To address a potential shift in metabolic flux towards acetate, we used acetate-route deficient *E. coli* cells, which lack the three major acetate pathway operons, ∆*ackA* ∆*poxB* ∆*pta* [45]. Exploiting our new plasmid-based *cydC* system, we examined *E. coli* ∆*ackA* ∆*poxB* ∆*pta* cells harboring both the isopentenol production pathway and plasmids with wild-type *cydC*, *cydC*-*D86G* or *cydC*-*D86V*. Similar to the results with limonene production in the presence of [EMIM]OAc, cells carrying wild-type *cydC* plasmid grew very poorly; we detected poor production of isopentenol in those cultures. In contrast, cells carrying the *cydC*-*D86G* plasmid produced robust titers of isopentenol in the presence of [EMIM]OAc (Fig. 5b), similar to published isopentenol production titers using this pathway in the absence of ILs [44]. Production titers were even higher in strains carrying a *cydC*-*D86V* plasmid, emphasizing the portability of this mutation to different strain backgrounds (Fig. 5b). From these results we conclude that *cydC*-*D86G* and *cydC*-*D86V* confer the robust capacity to generate two final products (limonene and isopentenol) under [EMIM]OAc stress, which to our knowledge is a first demonstration in the field.

## Discussion

ILs are a double-edged sword as a biomass pretreatment reagent: they are appealing because they can help efficiently extract sugar from recalcitrant lignocellulosic plant biomass, but often pose a detrimental effect on the downstream enzymes and microbial hosts for bioproduction of a target compound. In earlier reports, the improvement in IL tolerance has been shown to enhance product titers relative to strain productivity before engineering, but not at the levels possible under optimal growth conditions [14, 16, 46, 47]. This inability to fully recover production levels after engineering strains for IL tolerance indicates that the IL impacts cell growth and biosynthetic pathways separately; the biosynthetic pathway was still affected, even though overall growth under these conditions was restored. In combination, even minor reductions in strain productivity would limit the development of one-pot processes or even the use of unwashed pretreated biomass. The *E. coli cydC*-*D86G* chassis is an important step forward in the field, as it is both IL tolerant and retains the optimal efficiency of producing advanced biofuels. Future processes could utilize this mutation in *cydC* to develop one-pot methods with [EMIM]OAc pretreated lignocellulosic biomass, without compromising biofuel production.

We could not find the exact mechanism for the gain of function activity of the mutant *cydC*-*D86G* because *cydC* encodes an essential gene, a gene category that is challenging to utilize in metabolic engineering studies. Conditionally-essential gene collections are not yet widespread in bacteria, as has been completed for budding yeast [48]. The development of inducible CRISPRi libraries [49] will facilitate utilization of this gene reservoir and elucidate their cellular functions [50]. Spontaneous mutagenesis screens, such as via laboratory evolution, remain the standard for identifying advantageous gain-of-function mutations.

As evidenced by the breadth of functional categories implicated by our proteomics data, ionic liquids have a pleiotropic impact on bacterial systems. Similar results have been reported in other model organisms: in the yeast *S. cerevisiae*, [EMIM]OAc has been reported to cause a growth impact that exceeds that of [EMIM]Cl or NaOAc [51], and is thought to deform cell wall structure and limit oxygen transfer from the culture media into cells [35]. While we did not detect a decrease of the expression of genes related to fatty acid synthesis from this analysis, we did detect a five-fold decrease in the abundance of an essential gene, *plsB* (see Additional file 3: Table S2). PlsB is a critical for the downstream selection of fatty acids incorporated into membrane phospholipids, which could impact cell membrane structure or integrity [52]. However, the relationship between membrane lipid and protein composition and *cydC*-*D86G* remains to be unraveled.

**Fig. 6:**
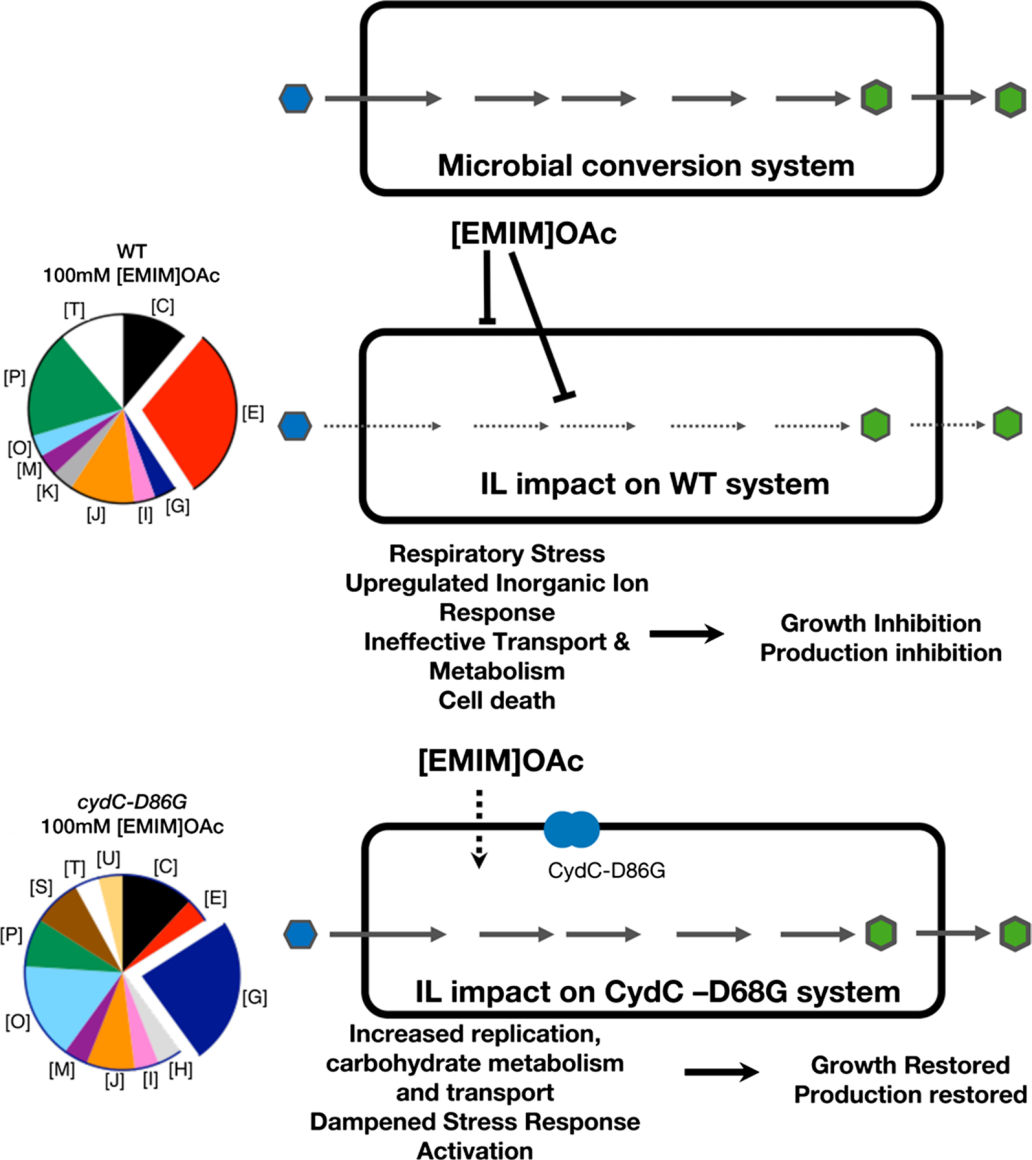
CydC modulates many cellular activities. Proposed role of *cydC* and ionic liquid tolerance. Proteomic analysis and experimental validation in *E. coli* deletion strains implicate a suite of pathways involved in the *cydC* response to [EMIM]OAc. In the *top panel*, cells express a heterologous gene pathway to produce a desired product, turning sugar (blue hexagon) into the target compound (green hexagon). The addition of the IL [EMIM]OAc in the *middle panel* has a broad impact on cellular physiology, impacting respiration, inorganic ion response factors, transporters, which ultimately inhibit cell growth and desired production. However, the *cydC*-*D86G* mutant in the *bottom panel* has a distinct proteomic response, and instead upregulates unique gene function categories, such as [U] intracellular trafficking and secretion and [G] carbohydrate metabolism and transport (see Fig. 4 for complete legend), ultimately restoring both cell growth and desired biofuel production

Previous studies have primarily resulted in the identification of native and non-native efflux pumps whose expression confers tolerance to [EMIM]OAc [14, 16, 53, 54]. In this study, we identify a new mechanism for bacterial resistance to [EMIM]OAc that addresses both the IL toxicity impact on cell growth and also protects the production pathway from inhibitory impact. It is unclear why mutations in candidates from two different protein families, specifically, efflux pumps and CydCD complex both conferred tolerance towards IL. However, both categories are fairly ubiquitous across bacteria and have broad substrate specificity [55]. We have demonstrated that our mutant, *cydC*-*D86G,* is a gain-of-function mutant and the over-expression of wild-type *cydC* alone does not confer tolerance to this IL in *E. coli*. While the complete mechanism for this IL tolerance remains to be understood, we speculate that this point mutation renders CydCD-complexes more promiscuous or active, but retains the minimum activities required for cellular viability. The mutant allele of *cydC* could result in CydCD-complexes with additional capacity to enhance multi-genic functions in carbohydrate transport and protein turnover/post translational modifications, without necessarily exporting a toxic metabolite from the cytoplasm (see Fig. 6). These gross changes could result in a distinct cytosolic response, blocking both the downstream impacts [EMIM]OAc on cellular processes as well as protecting the cell from the burden imposed by the mevalonate pathway for limonene or isopentenol production.

Our strategy to develop a microbial host chassis for the production of limonene in the presence of the IL, [EMIM]OAc, provides a template to obtain gain-of-function mutants applicable for cultivation under industrially-relevant conditions. By closely mimicking the conditions under which the target compound is produced, we obtained a single gain-of-function mutant in five independent trials in less than 40 h of incubation. In contrast to other laboratory evolution studies [15, 56, 57], we used an inhibitory concentration of the IL and included the potential burden of the limonene production plasmid in the screen. Future studies should consider the burden of a heterologous gene pathway as a fitness disadvantage when undertaking evolution studies.

This study exemplifies the use of interdisciplinary approaches to classical genetics and microbiology in conjunction with rational strain engineering to provide an elegant blueprint for optimizing microbial biofuel production under otherwise unfavorable culturing conditions. Heterologous gene pathways are a metabolic burden and have idiosyncratic impacts on native microbial metabolism. While *cydC*-*D86G* strains were already competent to produce d-limonene, the production of isopentenol in the presence of exogenous [EMIM]OAc required the use of acetate deficient strains to achieve the highest titer of isopentenol under these stress conditions. In retrospect, it is clear that a heterologous gene pathway could impact the native host metabolism, as we observed when we initially sought to produce isopentenol under IL stress conditions. A unified strategy for engineering microbial hosts will be critical in the development of promising non-model microorganisms in one-pot processes when final product toxicity is inhibitory [58, 59]. Metabolic flux models which incorporate data from all of these relevant impediments on central metabolism will advance our efforts to build robust cell factories.

## Methods

### Reagents, bacterial strains and plasmids

All chemicals used were reagent grade and purchased from Sigma-Aldrich (St. Louis, MO), unless otherwise noted. [EMIM]OAc, also known as [C_2_C_1_im]OAc, was purchased from Sigma-Aldrich and used as received. All strains and plasmids used in this work are listed at http://public-registry.jbei.org and are summarized in Additional file 5: Table S4. *E. coli* DH10B (Invitrogen, Carlsbad, CA) was used for cloning and manipulation of plasmids. Except for the experiments described in Table 1 from the Keio Collection, all IL tolerance assays were conducted with *E. coli* DH1. Mutants from the Keio collection were tested against the BW25113 wild-type strain. Plasmids were constructed using isothermal assembly with 40 bp overlapping homologous sequences and plasmid sequences were verified with restriction endonuclease digestion and Sanger Sequencing (Quintara Biosciences, Albany, CA). We defined the promoter sequence of the *cydDC* operon as the immediate upstream ~ 500 bp of *cydC.* Plasmid transformation was conducted as previously described [60] or else as expressly indicated.

### Generation of spontaneous mutants tolerant to [EMIM]OAc

*Escherichia coli* DH1 strains with or without specific genomic mutations and a limonene production plasmid were struck out from glycerol stocks and single colonies were used to inoculate 5 mL LB precultures. These individual precultures were then back-diluted into synthetic culture media with a 20% dodecane (Sigma Aldrich, St. Louis, MO) overlay and incubated at 30 °C until the cultures showed evidence of growth.

[EMIM]OAc tolerant *E. coli* strains were examined to determine if they contained heritable mutations as follows. Recovered mutants were washed three times in sterile deionized water and resuspended in fresh EZ Rich 1% d-glucose media. Approximately 100 µL of this resuspension was used to reinnoculate a fresh 25 mL culture lacking any ionic liquid. The optical density was measured over a 16-h period to ensure that at least eight doublings had occurred. Cells were then washed with sterile water as before and a similar back dilution into EZ Rich 1% d-glucose containing 100 mM [EMIM]OAc. The optical density was measured as before.

### Identification of mutant alleles by next generation illumina sequencing

Genomic DNA from candidate mutant strains was isolated using standard methods, and libraries were prepared for Illumina sequencing at the Joint Genome Institute (Walnut creek, CA, USA) as described by the manufacturer. In brief, DNA was randomly sheared into ~ 500 bp fragments to create an Illumina library. This library was sequenced on Illumina Miseq generating 150 bp paired end reads. Reads were aligned to the reference genome using BWA and putative SNPs and small indels were called using samtools and mpileup [61, 62]. Putative structural variants were called using a combination of BreakDancer (filtered to quality 90+), and Pindel [63, 64]. Basecall variants were called with CONV essentially as described by [65]. Candidate mutations were verified as absent in the parental *E. coli* genomic strain by Sanger Sequencing (Quintara Biosciences, South San Francisco CA).

### Strain culturing conditions

#### Limonene and isopentenol production

All production assays were conducted in the *E. coli* DH1 strain background. Strains containing the limonene production plasmid (JBEI-6409) were grown in EZ-Rich medium with the appropriate antibiotics to maintain plasmid selection, with glucose as the carbon source at a final concentration of 1% (10 g/L) (Teknova, Hollister, CA). 5 mL precultures were grown shaking (200 rpm) overnight at 30 °C and were used to inoculate starting 25 mL production cultures at an optical density of ~ 0.1 OD_600_. Cultures were grown for two doublings, to an OD_600_ of 0.4–0.6, at which expression of limonene production genes was induced with 15 μM β-d-1-thiogalactopyranoside (IPTG, Sigma Aldrich, St. Louis MO). A dodecane (Sigma Aldrich, St. Louis, MO) overlay (5 mL for 25 mL cultures) was applied at the time of IPTG induction, and was used to extract biosynthetically produced d-limonene. Limonene production was quantified 24 h or 48 h after IPTG induction using GC–MS as previously described [42].

The production of isopentenol was carried out essentially as described in Kang et al. [44] using the isopentenyl diphosphate bypass pathway to generate isopentenol. Briefly, cells of the indicated genotype were grown in EZ-Rich medium with the appropriate antibiotics to maintain plasmid selection, with glucose at the final concentration of 1% (10 g/L). Isopentenol production genes were induced with 500 μM IPTG. No dodecane overlay was applied. Isopentenol production was quantified 24 h or 48 h after IPTG induction using either GC–MS or GC–FID (Flame Ionization Detector) as previously described [43, 44].

#### Preparation of cells for proteomic analysis

To measure global protein abundance changes under IL stress conditions, the same strains used for limonene production in Fig. 4a were prepared exactly as described in 1% glucose EZ-Rich media, induced with 15 μM IPTG and a 20% dodecane overlay, in a 25 mL culture volume. Approximately 3 × 10^9^ cells were harvested by centrifugation at 14,000*g* for 3 min and stored at − 80 °C until analysis. Four biological replicates across two trials were used for the shotgun proteomics analysis. Another 3 × 10^9^ cells were harvested by centrifugation and prepared for peptide analysis. Samples for peptide analysis were also prepared in biological quadruplicate.

### Assessment of gene essentiality by plasmid shuffle

Wild-type *E. coli* DH1 strains were sequentially transformed with pKD46 [*P*_*bad*_-*gam*-*beta*-*exo AmpR*] followed by pTE50 [*sacB P*_*CYDD*_-*cydDC KanR*] using electroporation (Protocol No. 26, page 1.119) [29, 66]. Briefly, a fresh colony from a glycerol stock was inoculated into a 50 mL LB 1% arabinose (Sigma-Aldrich, St. Louis MO) preculture, and grown to an optical density of ~ 0.4 at 600 nm. Cells were harvested by centrifugation at 4000*g* for 10 min, washed three times with 50 mL cold dH_2_O, and prepared for transformation via electroporation by resuspending the washed cell pellet in 1 mL of 10% glycerol. Cells were transformed with approximately 100 ng of a PCR fragment containing the Hygromycin B drug cassette [67] flanked on either side with 3 kb of homology to the upstream or downstream regions immediately adjacent to *cydC.* After DNA electroporation, cells were outgrown for 3 h in SOC media (New England Biolabs, Ipswich, MA) supplemented with 0.5% arabinose and plated onto LB plates containing 70 µg/mL Hygromycin B (Roche Life Sciences, Indianapolis IN). Hygromycin resistant clones were observed after 1 day post plating after incubation at 30 °C. Genomic deletions were verified by colony PCR to ensure replacement of the *cydC* locus with the hygromycin cassette using primers flanking the deletion cassette that would not amplify the plasmid-borne *cydC*. Spontaneous loss of pKD46 was confirmed after incubating validated clones at 42 °C for 1 day on LB plates and testing single colonies for the ability to grow on LB ampicillin plates. Due to the extended outgrowth phase used in this protocol, three independent electroporation reactions were conducted in parallel, and multiple validated colonies from different transformations were used for subsequent experiments.

Following strain generation, strains harboring either the wild-type *cydC* locus or the genomic deletion as well as pTE50 [*sacB P*_*CYDD*_-*cydDC KanR*] were struck to single colonies from glycerol stocks. Single colonies were inoculated into LB precultures for 24 h to allow for spontaneous plasmid loss in the population. After outgrowth, cells were serially diluted in a 96 well microtiter plate and 3 μL of each tenfold dilution was plated on solid agar media containing LB supplemented with or without 10% sucrose. Photomicrographs of each plate were taken approximately 1 day post plating.

### Peroxide sensitivity assay

To determine the correlation between [EMIM]OAc resistance and hydrogen peroxide resistance, candidate deletion strains from the *E. coli* BW25113 background isolated from the Keio collection were selected for analysis [29]. Genomic deletions were confirmed by colony PCR using primers flanking the kanamycin drug cassette and the hydrogen peroxide assay was conducted as previously described [68]. In brief, fresh overnight cultures of the appropriate genotype were back diluted into 96 well microtiter dishes containing LB media supplemented with or without 30 mM hydrogen peroxide (Sigma Aldrich, St. Louis, MO). Growth was monitored in a BioTek (Winooski, VT) Synergy plate reader by measuring optical density at OD_600_ at 15-min intervals. All experiments were conducted in triplicate and repeated at least twice over the course of several weeks.

### Proteomics

Samples prepared for shotgun proteomic experiments were analyzed by using an Agilent 6550 iFunnel Q-TOF mass spectrometer (Agilent Technologies, Santa Clara, CA) coupled to an Agilent 1290 UHPLC system as described previously [69]. Twenty (20) μg of peptides were separated on a Sigma–Aldrich Ascentis Peptides ES-C18 column (2.1 mm × 100 mm, 2.7 μm particle size, operated at 60 °C) at a 0.400 mL/min flow rate and eluted with the following gradient described previously [69]. The acquired data was exported as MGF files and searched against the latest *E. coli* (strain K12) protein database with Mascot search engine version 2.3.02 (Matrix Science). The resulting search results were filtered and analyzed by Scaffold v 4.3.0 (Proteome Software Inc.). Normalized spectra counts of identified proteins were exported for quantitative analysis and selection of target proteins, which were significantly altered between experimental groups. These significantly altered proteins were classified into COG terms using eggnog 4.5 and are accessible at https://goo.gl/GgQGns.

A targeted SRM method was developed to target peptides of these proteins by using an in-house build *E. coli* spectral library. The SRM targeted proteomic assays were performed, as described previously [39], on an Agilent 6460 QQQ mass spectrometer system coupled with an Agilent 1290 UHPLC system (Agilent Technologies, Santa Clara, CA). Peptides were separated on an Ascentis Express Peptide C18 column [2.7-mm particle size, 160-Å pore size, 5-cm length × 2.1-mm inside diameter (ID), coupled to a 5-mm × 2.1-mm ID guard column with the same particle and pore size, operating at 60 °C; Sigma-Aldrich] operating at a flow rate of 0.4 mL/min via the following gradient: initial conditions were 98% solvent A (0.1% formic acid), 2% solvent B (99.9% acetonitrile, 0.1% formic acid). Solvent B was increased to 35% over 6.5 min, and was then increased to 80% over 1.5 min, and held for 1.5 min at a flow rate of 0.6 mL/min, followed by a ramp back down to 2% B over 0.5 min where it was held for 1 min to re-equilibrate the column to original conditions. The data were acquired using Agilent MassHunter version B.08.02. Acquired SRM data were analyzed by Skyline software version 3.70 (MacCoss Lab Software). The SRM methods and data are available at Panoramaweb [70] https://goo.gl/GgQGns.

### Quantification of [EMIM]OAc in growth media by fourier-transform infrared spectroscopy (FT-IR) and high performance liquid chromatography–liquid chromatography mass spectrometry (HPLC–LCMS)

Quantification of [EMIM]OAc by FTIR was done essentially as described in [71] using cells grown in LB media. Briefly, quantitative measurement of the imidazolium-based ionic liquid was interrogated using the unique peak height at 1170.59 cm^−1^ corresponding to the presence of [EMIM]OAc. A standard curve was generated using commercially synthesized [EMIM]OAc. LC–MS analysis of [EMIM]^+^ was carried out with a method similar to the one described by Bokinsky et al. that was used to measure amino acids [72]. The only differences were the column compartment temperature, which was set to 20 °C, and the LC gradient elution method, described as follows: linearly increased from 90% B to 70% B in 4 min, held at 70% B for 1.5 min, decreased from 70% B to 40% B in 0.5 min, held at 40% B for 2.5 min, increased from 40% B to 90% B in 0.5 min, and held at 90% B for 2 min. The flow rate was held at 0.6 mL/min for 6.5 min, increased from 0.6 mL/min to 1 mL/min in 0.5 min, and held at 1 mL/min for 4 min. The total LC run time was 11 min.

## Additional files

**Additional file 1: Figure S1.** Viability of *E. coli DH1 pTRC-cydC* strains. *E. coli* DH1 cells over-expressing *P*_*trc*_-*cydD-cydC* (pTE42) or *P*_*trc*_-*cydD-cydC-D86G* (pTE43) were inoculated with concentrations of the inducer IPTG ranging from 0-32 µM IPTG (“Methods”). Optical density was monitored at 15-minute time points over a 24 hour timeframe. Optical density is plotted on a log_10_ scale. *E. coli DH1* cells overexpressing *cydC* were slow growing at inducer concentrations from 4-16 µM IPTG and inviable at concentrations above 32 µM IPTG. Cells overexpressing *cydC-D86G* exhibited less of a decrease of doubling time with IPTG concentrations up to 32 µM IPTG, but were also inviable at 125 µM IPTG. A comparison of doubling time as a function of IPTG concentration for both strains is provided in the bottom panel. **Figure S2.** *E. coli* DH1 *cydC-D86G* Is Also Tolerant to the IL Ethanolamine Acetate (EOA). Wild type and *cydC-D86G* cells were prepared for growth as in Figure 3 (“Methods”) and grown in the presence or absence of the IL ethanolamine acetate ([EOA]OAc) exactly as described in Figure 1. Both wild-type and *cydC-D86G* strains had identical growth curves at 30mM and 300mM EOA. Optical density is plotted on a log_10_ scale. **Figure S3.** Overexpression of *cydAB* Does Not Improve Tolerance to [EMIM]OAc. Related to Figure 3. **(A, B)** *E coli* BW25113 cells harboring a P_LacUV5_-*cydAB* overexpression cassette and a genomic deletion at *oppF* were prepared for exogenous [EMIM]OAc treatment as described in Figure 3. Cells of the genotype indicated (black circles, WT; red squares, ∆*oppF*) were tested with or without 50 mM [EMIM]OAc (panel A, with [EMIM]OAc; panel B, without [EMIM]OAc) and with or without 200 µM IPTG (panel A, without IPTG; panel B, with IPTG). Optical density is plotted on a log_10_ scale. **(C)** Increased gene dosage of *cydC* is sufficient to confer tolerance to [EMIM]OAc in *E. coli* BW25113 strains. Plasmids harboring either *cydC* (pTE50) or *cydC-D86G* (pTE88) or an empty vector control (pK18mobsacB) were transformed into *E. coli* BW25113. Cells of the indicated genotype were then 10-fold serially diluted onto solid LB agar media supplemented with or without 100 mM [EMIM]OAc. LB plates were photographed 1 day after growth at 30 °C; LB 100 mM [EMIM]OAc plates were photographed 4 days after growth at 30 °C. A subtle increase in tolerance against exogenous [EMIM]OAc was observed in strains carrying pTE88 (*cydC-D86G)* over pTE50 (*cydC*), but both strains were more resistant compared to the empty vector control. **Figure S4.** Exogenous [EMIM]OAc Concentration Does Not Change in *cydC-D86G* Strains. Wild-type *E. coli* DH1 and *E. coli* DH1 *cydC-D86G* strains were grown as described in Figure 4. (A) Measurement of [EMIM]OAc in growth media by FTIR analysis. After ~20 hours of growth, the cell culture media was subjected to room temperature FTIR analysis. Left panel, standard curve using serial dilutions of [EMIM]OAc standard. Right panel, quantification of [EMIM]OAc in biological triplicate samples. No significant difference in [EMIM]OAc concentration was detected between wild-type, *cydC-D86G*, or growth media alone. (B) Measurement of [EMIM]OAc in growth media by HPLC-MS. Left panel, standard curve using serial dilutions of [EMIM]OAc. Right panel, quantification of [EMIM]OAc by HPLC-LCMS in biological triplicate samples. **Figure S5.** Specific production of limonene and isopentenol normalized to cell density (biomass). Samples prepared and described in Figure 5 were normalized to cell density, as measured as optical density; Absorbance at 600 nm.

**Additional file 2.** Phylogenetic analysis of *cydC* and *cydB* genomic co-occurrence in bacterial genomes.

**Additional file 3.** Shotgun proteomics raw data for *cydC*-D86G and control strains.

**Additional file 4.** Targeted MRM proteomics raw data for *cydC*-D86G and control strains.

**Additional file 5.** Strains and plasmids used in this study.

## Abbreviations

IL: ionic liquid
[EMIM]OAc and [C_2_C_1_im]OAc: 1-ethyl-3-methyl-imidizolium acetate
IPTG: isopropyl β-d-1-thiogalactopyranoside
COG: clusters of orthologous groups
FID: flame ionization detector
