# Supplementary material for: Restoration of biofuel production levels and increased tolerance under ionic liquid stress is enabled by a mutation in the essential Escherichia coli gene cydC
Source: Microb Cell Fact. 2018 Oct 8;17:159. doi: 10.1186/s12934-018-1006-8 (PMC6174563; doi:10.1186/s12934-018-1006-8)
Supplement: Supplementary file 1 — Additional file 1: Figure S1. Viability of E. coli DH1 pTRC-cydC strains. E. coli DH1 cells over-expressing Ptrc-cydD-cydC (pTE42) or Ptrc-cydD-cydC-D86G (pTE43) were inoculated with concentrations of the inducer IPTG ranging from 0-32 µM IPTG (“Methods”). Optical density was monitored at 15-minute time points over a 24 hour timeframe. Optical density is plotted on a log10 scale. E. coli DH1 cells overexpressing cydC were slow growing at inducer concentrations from 4-16 µM IPTG and inviable at concentrations above 32 µM IPTG. Cells overexpressing cydC-D86G exhibited less of a decrease of doubling time with IPTG concentrations up to 32 µM IPTG, but were also inviable at 125 µM IPTG. A comparison of doubling time as a function of IPTG concentration for both strains is provided in the bottom panel. Figure S2. E. coli DH1 cydC-D86G Is Also Tolerant to the IL Ethanolamine Acetate (EOA). Wild type and cydC-D86G cells were prepared for growth as in Figure 3 (“Methods”) and grown in the presence or absence of the IL ethanolamine acetate ([EOA]OAc) exactly as described in Figure 1. Both wild-type and cydC-D86G strains had identical growth curves at 30mM and 300mM EOA. Optical density is plotted on a log10 scale. Figure S3. Overexpression of cydAB Does Not Improve Tolerance to [EMIM]OAc. Related to Figure 3. (A, B) E coli BW25113 cells harboring a PLacUV5-cydAB overexpression cassette and a genomic deletion at oppF were prepared for exogenous [EMIM]OAc treatment as described in Figure 3. Cells of the genotype indicated (black circles, WT; red squares, ∆oppF) were tested with or without 50 mM [EMIM]OAc (panel A, with [EMIM]OAc; panel B, without [EMIM]OAc) and with or without 200 µM IPTG (panel A, without IPTG; panel B, with IPTG). Optical density is plotted on a log10 scale. (C) Increased gene dosage of cydC is sufficient to confer tolerance to [EMIM]OAc in E. coli BW25113 strains. Plasmids harboring either cydC (pTE50) or cydC-D86G (pTE88) or an empty vector control (pK18mobs [file 12934_2018_1006_MOESM1_ESM.pptx]

## Slide 1
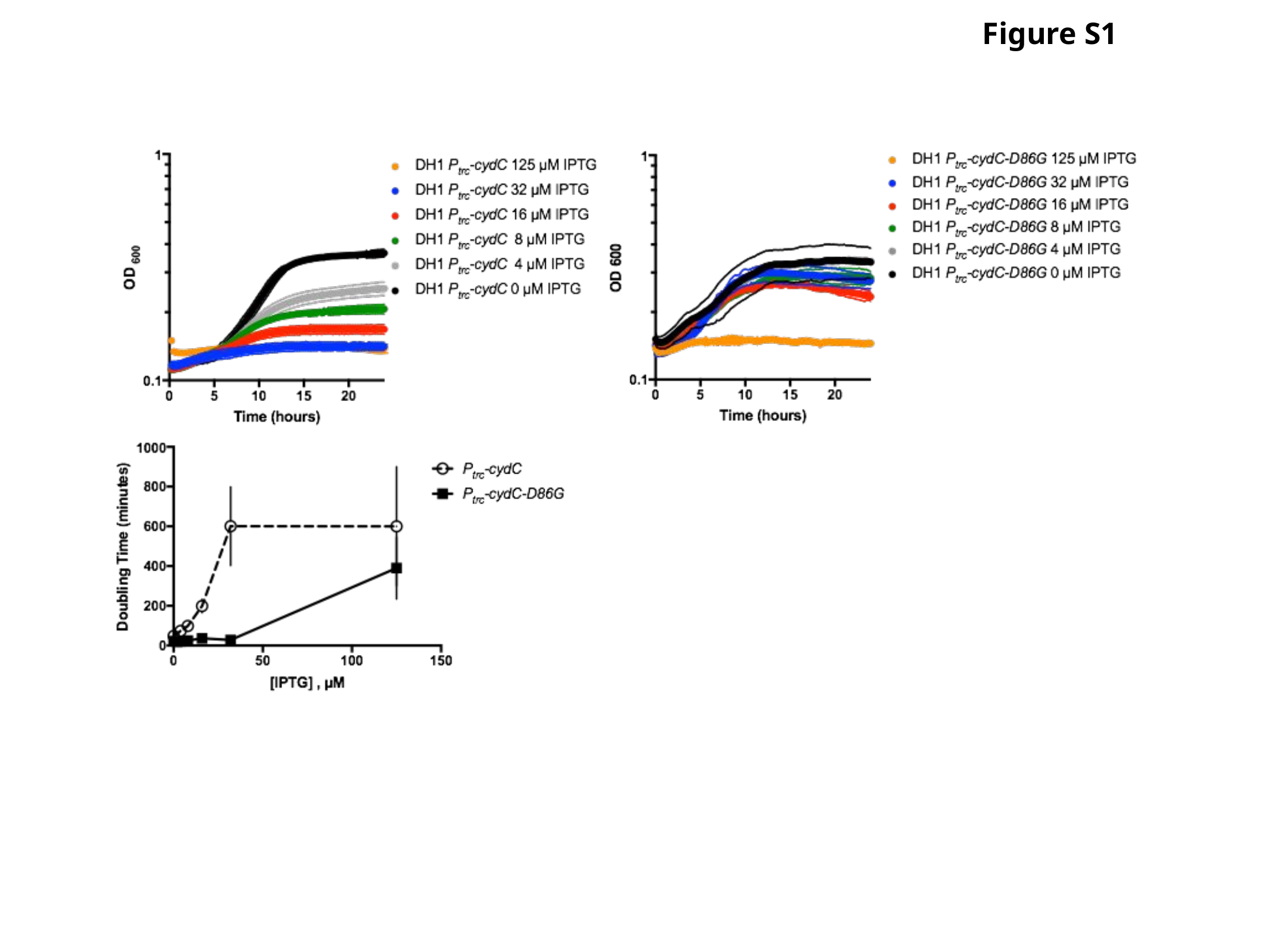

Figure S1

## Slide 2
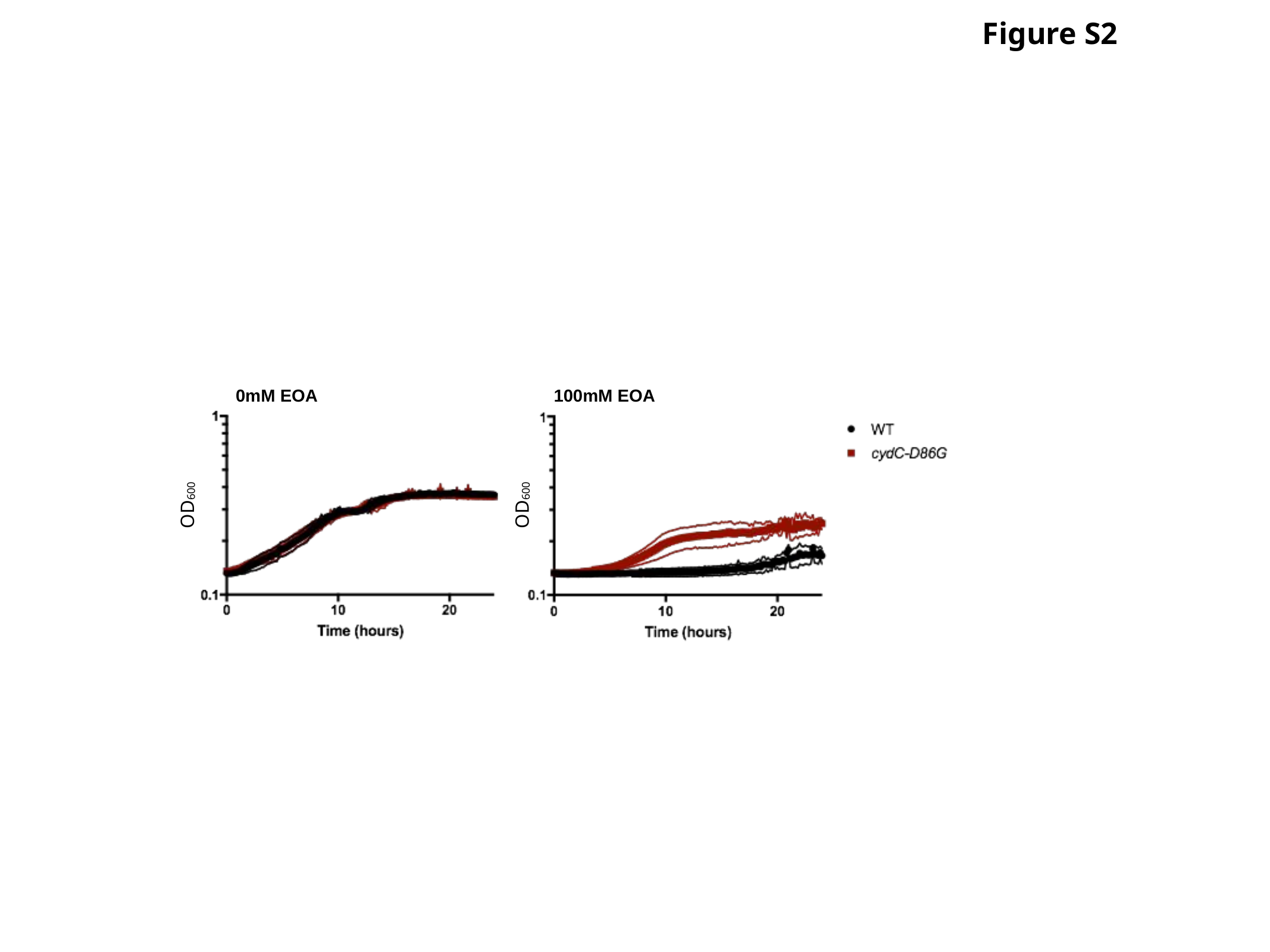

Figure S2
0mM EOA
100mM EOA
OD600
OD600

## Slide 3
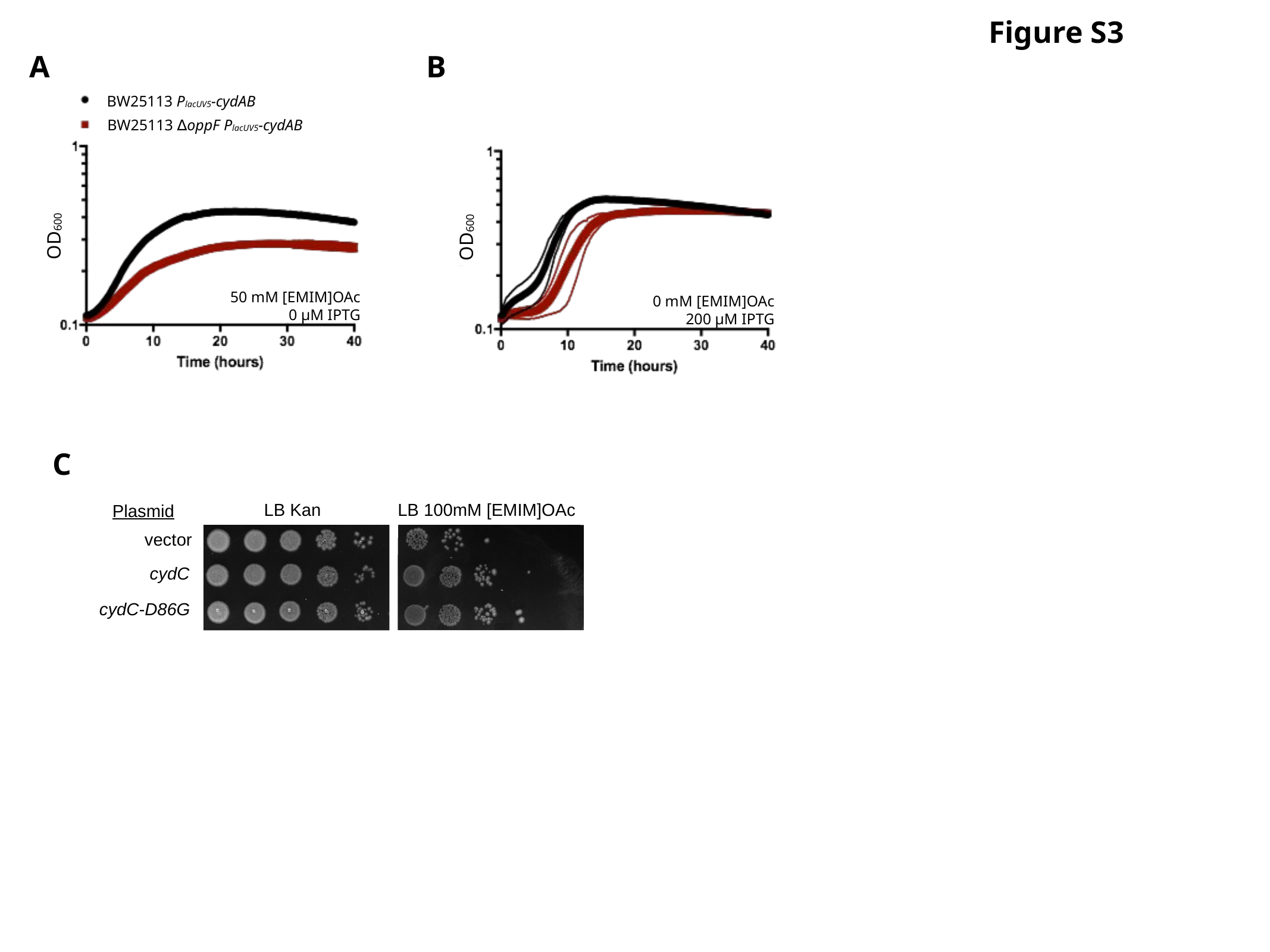

Figure S3
A
B
BW25113 PlacUV5-cydAB
BW25113 ∆oppF PlacUV5-cydAB
OD600
50 mM [EMIM]OAc
0 µM IPTG
OD600
0 mM [EMIM]OAc
200 µM IPTG
C
LB Kan
LB 100mM [EMIM]OAc
Plasmid
vector
cydC
cydC-D86G

## Slide 4
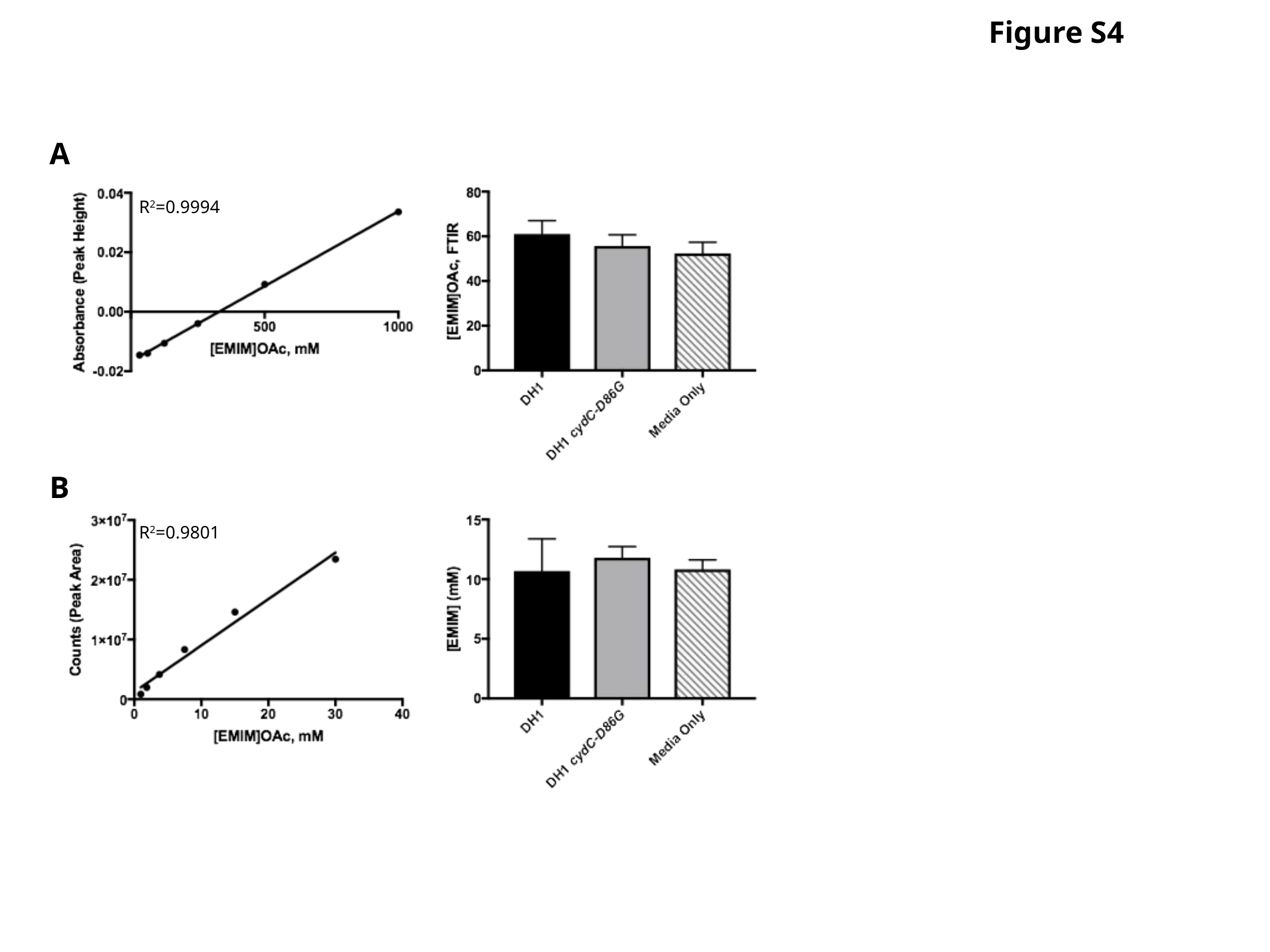

Figure S4
A
R2=0.9994
B
R2=0.9801

## Slide 5
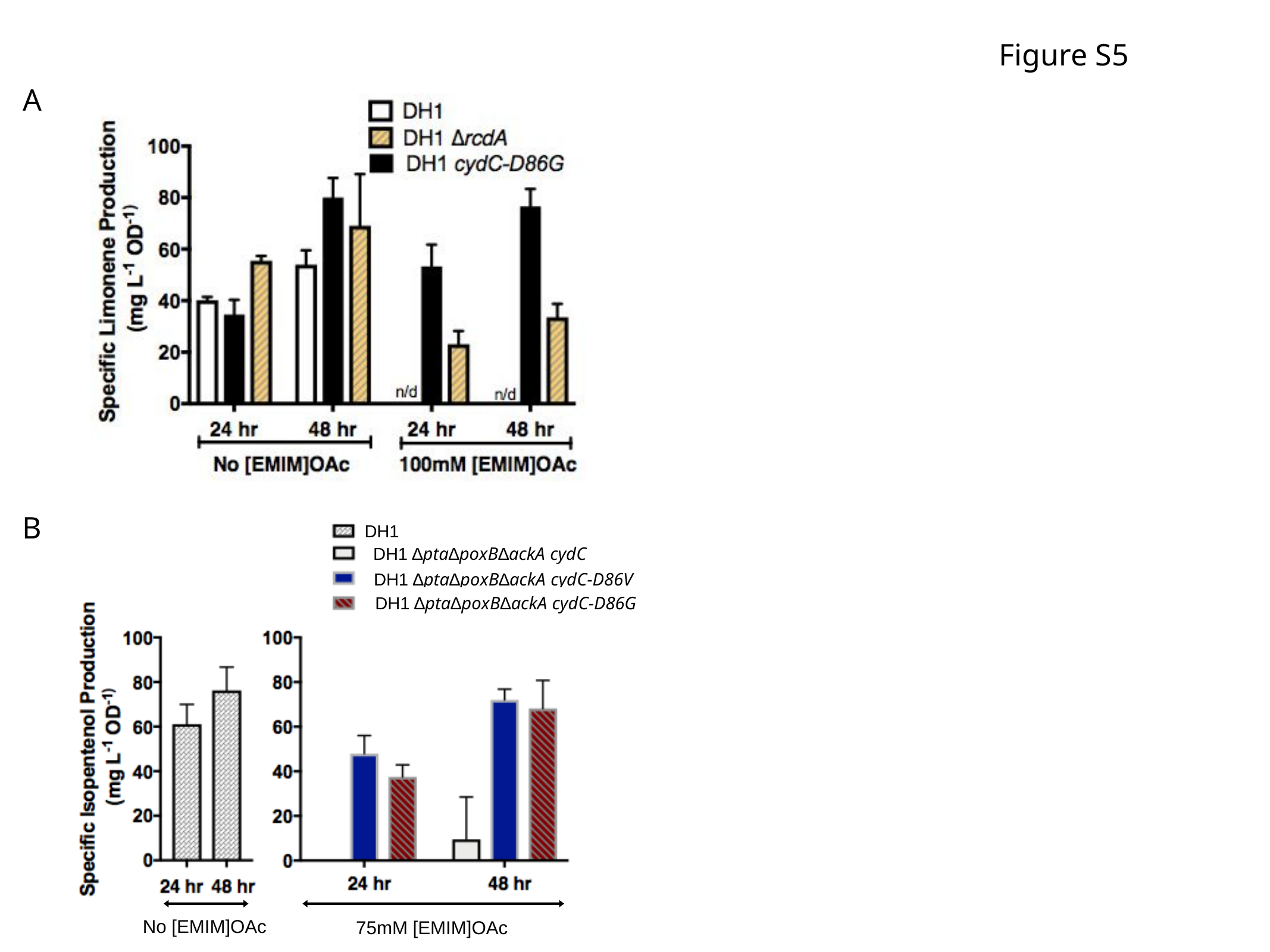

Figure S5
A
B
DH1
DH1 ∆pta∆poxB∆ackA cydC
DH1 ∆pta∆poxB∆ackA cydC-D86V
DH1 ∆pta∆poxB∆ackA cydC-D86G
No [EMIM]OAc
75mM [EMIM]OAc
