# Supplementary material for: Restoration of biofuel production levels and increased tolerance under ionic liquid stress is enabled by a mutation in the essential Escherichia coli gene cydC
Source: Microb Cell Fact. 2018 Oct 8;17:159. doi: 10.1186/s12934-018-1006-8 (PMC6174563; doi:10.1186/s12934-018-1006-8)
Supplement: Supplementary file 5 — Additional file 5. Strains and plasmids used in this study. [file 12934_2018_1006_MOESM5_ESM.docx]

**Strain table**

*Production Strains*:

K-2151 DH1

JBEI-18791 DH1 *cydC-D86G*

JBEI-18744 DH1 *∆cydC*::*HphMx6* [*pK18mobsacB-kan-P_cydD_-cydD-cydC*]

JBEI-3606 DH1 ∆*ackA*∆*poxB*∆*pta*

JBEI-13314 DH1 *∆rcdA*

*Deletion Strains* (Baba et al)

| ∆*yihL* | JW3837 |
| --- | --- |
| ∆*pykA* | JW1843 |
| ∆*uspG* | JW0600 |
| ∆*manX* | JW1806 |
| ∆*acnA* | JW1268 |
| ∆*nuoC* | JW5375 |
| ∆*zapB* | JW3899 |
| ∆*ybgL* | JW0703 |
| ∆*cysI* | JW2733 |
| ∆*grxB* | JW1051 |
| ∆*slp* | JW3474 |
| ∆*ydiH* | JW1675 |
| ∆*osmC* | JW1477 |
| ∆*pspA* | JW1297 |
| ∆*cysH* | JW2732 |
| ∆*fbaB* | JW5344 |
| ∆*ugpB* | JW3418 |
| ∆*cysN* | JW2721 |
| ∆*katE* | JW1721 |
| ∆*adhP* | JW1474 |
| ∆*rcdA* | JW5114 |
| ∆*emrE* | JW0531 |

*Plasmids*

JBEI-6409 Limonene production plasmid

JBEI-9321 Isopentenol production plasmid

ATCC-87097 pK18mobsacB

JBEI-18790 pTE50 *pK18mobsacB-kan-P_cydD_-cydD-cydC*

JBEI-18789 pTE88 *pK18mobsacB-kan-P_cydD_-cydD-cydC-D86G*

JBEI-18787 pTE100 *pK18mobsacB-kan-P_cydD_-cydD-cydC-D86V*

JBEI-18788 pTE42 *P_trc_-cydD-cydC*
JBEI-18742 pTE43 *P_trc_-cydD-cydC-D86G*
